# *Francisella* induced microparticulate caspase-1/gasdermin-D activation is regulated by NLRP3 independent of Pyrin

**DOI:** 10.1371/journal.pone.0209931

**Published:** 2018-12-31

**Authors:** Srabani Mitra, Erin Dolvin, Karthikeyan Krishnamurthy, Mark D. Wewers, Anasuya Sarkar

**Affiliations:** 1 Department of Physiology and Cell Biology, The Ohio State University, Columbus, OH, United States of America; 2 Department of Internal Medicine, The Ohio State University, Columbus, OH, United States of America; Louisiana State University, UNITED STATES

## Abstract

Although the study of pathogen sensing by host defense systems continues to uncover a role for inflammasome components specific to particular pathogens, gaps remain in our knowledge. After internalization, *Francisella* escapes from the phagosome in mononuclear cells and is thought to be detected by intracellular pathogen-response-receptors pyrin and Aim2 in human and murine models, respectively. However, it remains controversial as to the role of pyrin in detecting *Francisella*. Our current work aims to study the contribution of inflammasome sensor, Pyrin in regulating microparticulate caspase-1/GSDM-D activation by *Francisella*. Our findings suggest that NLRP3 is central to the activation/release of active caspase-1/GSDM-D encapsulated in microparticles (MP) by *Francisella*. We also provide evidence that this regulation is independent of pyrin, implicated in sensing cytosolic *Francisella* in NLRP3-/- conditions where endogenous Pyrin is present. Absence of NLRP3 completely abrogated *Francisella* mediated MP caspase-1/GSDM-D activation and release both before and after internalization of the pathogen. However, deletion of pyrin not only enhanced both LPS and *Francisella* mediated MP active caspase-1/GSDM-D release, but pyrin overexpression resulted in a reduction of inflammasome activation and release; suggesting an inhibitory role of pyrin in LPS and *Francisella* mediated MP responses. This NLRP3 dependence and inhibitory effect of pyrin correlated with cytokine release as well. These observations also correlated with MPs ability to induce cell death; as LPS and *Francisella*-induced MPs from pyrin-deficient cells were more potent than wild-type monocytes whereas, NLRP3-/- MPs failed to induce cell death. Taken together, we report that NLPR3 not only mediates *Francisella* induced cytokine responses, but is also critical for cytokine-independent microparticle-induced inflammasome activation and endothelial cell injury independent of pyrin.

## Introduction

Inflammasome activation forms one of the first lines of defense in the innate immune system to fight pathogens [1]. PAMPs (pathogen associated molecular patterns) or DAMPS (danger associated molecular patterns) sensed by different PRR (pathogen recognition receptor) leads to the induction of inflammasome response mediated caspase-1 activation and tissue damage [1,2]. Caspase-1 activation is central to every inflammasome activation upon sensed by the pathogen receptor NLR family [3]. NLRP3, one of the most extensively studied receptors, can be activated by a wide variety of PAMPs such as nigericin and DAMPS like ATP and MSU (monosodium urate) crystals [4–8]. Upon recognition, NLRP3 is known to induce inflammasome activation, thereby facilitating release of pro-inflammatory cytokines, IL-1β and IL-18 to combat infection. Microparticles are small membrane coated vesicles that are released from cells upon activation or apoptosis. Microparticles have been described to be critical for the release of active inflammasome in pathological states. Prior work from our laboratory and others have described the role of microparticulate active caspase-1 and GSDM-D, as well as NLRP3 in regulating cell fate upon inflammasome activation [9–13].

*Francisella novicida* is genetically close to *Francisella tularensis*, a gram-negative bacterium which causes a disease called tularemia [14]. *Francisella* belongs to a select group of bacteria, including *Listeria*, *Shigella* etc which proliferate within the host cell by evading the immune responses of pathogen defense. *Francisella* infection induces fever and cell death along with secretion of pro-inflammatory cytokine, IL-1β via inflammasome activation [15,16]. Although innate immunity against *Francisella* has been described to be dependent on the ASC/caspase-1 axis [16], controversy remains as to the specific pathogen receptors for *Francisella*. Prior work from others have described that type 1 interferon signaling is necessary for *Francisella* mediated inflammasome activation, cytokine release and cell death [17]. It has also been reported that the pathogen is recognized before its internalization by multiple pathways, including NLRP3 leading to IL-1β synthesis [18]. Upon internalization and escape from phagosome, it is thought that this response is mostly regulated by pyrin in human mononuclear cells [19] and Aim2 in murine models [20]. However, the role of pyrin in regulating *Francisella* mediated inflammasome responses has been a subject of controversy since pyrin exhibits both anti-inflammatory effects, via inhibition of inflammasome mediated IL-1β activation [21–23] as well as pro-inflammatory effects, via activation of the inflammasome [19,24,25].

In order to determine the contribution of NLRP3 vs pyrin in regulating microparticulate inflammasome complex activation and responses by *Francisella*, we utilized the canonical LPS model of NLRP3 inflammasome signaling. Our findings suggest that NLRP3 is central to regulation of microparticulate inflammasome activation and release of active caspase-1 and GSDM-D by *Francisella* from human monocytic cells. In fact, the sensing and uptake of *Francisella* by monocytes was independent of pyrin, a protein that has previously been implicated in sensing intracellular *Francisella*. Moreover, using pyrin overexpression models, we demonstrate that overexpression of pyrin inhibited microparticulate inflammasome activation and release of active caspase-1 and GSDM-D by both LPS and *Francisella*, possibly by virtue of its anti-inflammatory role.

## Materials and methods

### Cells, bacterial strains and reagents

Highly purified Lipopolysaccharide (LPS) from *Escherichia coli* strain 0111:B4 was purchased from Invivogen (San Diego, CA). *Francisella novicida* strain U112 (JSG2401) was provided by M. Gavrilin (The Ohio State University, Columbus, OH). Bacteria were grown on chocolate II agar plate (BD Biosciences, Sparks, MD) at 37°C, harvested and re-suspended in cell culture medium without antibiotics before adding to cells. RPMI 1640 was purchased from Mediatech, Inc. (Manassas, VA); phosphate buffered saline (PBS) from Life Technologies (Grand Island, NY) and fetal bovine serum (FBS) from Atlanta Biologicals (Atlanta, GA). All medium were supplemented with 10% heat-inactivated FBS and 1% penicillin-streptomycin (Invitrogen Life Technologies). Mouse anti-GSDM-D was obtained from Abnova (Taipei, Taiwan). Antibodies for caspase 1 (captures both p45 kD and p20 active forms in human) and Pyrin antibodies were generated in house (Covance, Princeton, NJ). Other antibodies used in this study include NLRP3 (Adipogen, San Diego, CA) and Hsp90 (Cell Signaling, Danvers, MA). All other reagents were obtained from Sigma-Aldrich (St. Louis, MO) unless otherwise specified.

### Cell culture

Human THP1 monocytic cells obtained from American Type culture collection. THP1/ Cas9, THP1 Cas9/ Pyrin KO, THP1 Cas9/NALP3 KO and THP1 Cas9/ Pyrin KO/KI cells were generated by Dr. Seth Masters, WEHI, Australia. THP EGFP-Pyrin and THP EGFP-Pyrin overexpressed were generated by Dr. Mikhail Gavrilin (The Ohio State University). Cells were routinely cultured in RPMI 1640 supplemented with 10% fetal bovine serum (FBS) at 37°C in a humidified CO_2_ incubator. Some cells were cultured for 4h or overnight in the presence or absence of LPS (1μg/ml) or 25 MOI *Francisell*a. Primary human pulmonary microvascular endothelial cells (HPVECs) were obtained from Lonza (Walkersville, MD). They were maintained in EGM2 MV Bulletkit System (Lonza) as per manufacturer’s protocol. Co-culture experiments of HPVECs with microparticles were performed in RPMI medium with 10% FBS throughout the experiments.

### Microparticle isolation

Microparticle (MP) isolation was performed following our published protocol [9]. Briefly, culture media from cells stimulated or not with LPS (1μg/ml) or 25 MOI *Francisella* for different time points were collected and centrifuged at 2000 g for 5 min and 16,000 g for 5 min to remove floating cells and cell debris. The culture media was further centrifuged at 100,000 g for 1h to isolate microparticles. Microparticles were then re-suspended in lysis buffer or RPMI based on the requirement of the experiment. Functional studies were then performed with the pelleted MPs by subjecting them to immunoblotting, or co-culture assays with endothelial cells (HPVECs).

### Microparticle quantification

MPs isolated from THP1 monocytic cells treated with LPS (LPS MP) or left untreated (control MP) were subjected to quantification analysis for normalization purposes. First, total proteins were measured from the MPs. MPs were then subjected to quantification using the NanoSight technology following the company manual. The Malvern NanoSight range of instruments utilizes Nanoparticle Tracking Analysis (NTA) to characterize nanoparticles from 10nm-2000nm in solution. Each particle is individually but simultaneously analyzed by direct observation and measurement of diffusion events. Based on our analysis, control and LPS MPs when normalized using protein quantification showed similar number of particles. Based on this observation, all MPs were analyzed based on protein normalization and added to endothelial cells for cell death assays.

### Cell death assays

Endothelial cell death assay was performed as described earlier [22]. Briefly endothelial cells cultured at a confluency of 60–70% were subjected to microparticles released from THP1. Cell death analysis of HPVECs were performed by MTS assay according to manufacturer’s protocol (Promega, Madison, WI). The amount of colored product formed is directly proportional to the number of living cells in culture measured at 490nm. In this study the endothelial cells seeded in a 12 well tissue culture plate were treated with microparticles in a CO_2_ incubator at 37°C overnight. Medium was removed the following day, cells washed three times with RPMI medium containing 10% FBS. The MTS reagent was added to the cells at a ratio of 1:5 (Reagent mixture: PBS+4.5g/L glucose) and incubated for 1h at 37°C in a CO_2_ incubator. The absorbance was measured in a plate reader 490nm (Perkin Elmer 2030 Multilabel Reader, Shelton CT). Cell death was also analyzed by Trypan Blue spectrometric assay as previously described [10]. Briefly, supernatants from endothelial cells treated with MPs were spun at 16000g for 5 min to collect the dead floating cells after treatment. Cells were then washed 2X with PBS and incubated with 0.05% Trypan Blue in PBS at 37°C for 15 min. Cells were spun at 16000g and dye containing PBS removed. The residual dye was then removed by washing (2 X 1ml) and centrifuged each time at 16000g. The cells were lysed with 200ul of lysis buffer (25mM Tris-HCl pH 7.4, 120mM NaCl and 1% SDS). Finally, the solution was transferred to a 96 well culture dish and analyzed spectrophotometrically at 562nm.

### Immunoblotting

Culture media was removed from cells and differentially centrifuged to collect cell pellets and microparticles. Cell pellets or microparticles were lysed in lysis buffer (50mM Tris-HCl pH8.0, 125mM NaCl, 10mM EDTA, 10mM sodium fluoride, 10mM sodium pyrophosphate and 1% Triton X-100 containing protease inhibitor cocktail from Sigma and 50μM N-methoxysuccinyl–Ala-Ala-Pro-Val chloromethylketone). The protein concentration in the cell lysates and microparticles was determined by Dc Lowry protein assay reagent (Bio Rad). Equal amounts of total protein were resolved by SDS-PAGE and transferred to PVDF membrane. The membrane was then blocked with 10% nonfat dry milk in TBST (25mM Tris-HCl pH 7.5, 150mM NaCl, .1% Tween 20) for 2h at room temperature. The membranes were then probed with primary antibodies as indicated followed by peroxidase conjugated secondary antibodies. Protein bands were visualized by enhanced chemiluminescence (ECL, GE Healthcare).

### ELISA

Sandwich ELISAs were developed in our laboratory to detect mature IL-1β. Briefly, anti-human mouse monoclonal antibody (clone 2805, R&D Systems) was used as a coating antibody and a rabbit polyclonal mature IL-1β (raised against entire 17 KDa mature IL-1β) was used respectively. Horseradish peroxidase (Bio Rad)–conjugated goat anti rabbit antibody was used as a developing antibody. Released IL-18 was quantified by sandwich ELISA using MBL antibodies as described [26]. Briefly anti-human IL-18 mouse IgG2A (MBL International) was used as the coating antibody and a rat Biotin labelled anti human IL-18 (MBL International) was used respectively. Streptavidin- HRP (eBiosciences) was used as a developing antibody. Plates were read on a Perkin Elmer 2030 Victor Multilabel Reader, absorbance at 450nm.

### Statistical analysis

Data are represented as the mean ± standard error of the mean (SEM) from at least three independent experiments. p<0.05 was considered to represent statistical significance.

## Results

### NLRP3 regulates active caspase-1 and GSDM-D release by *Francisella*

**Fig 1:**
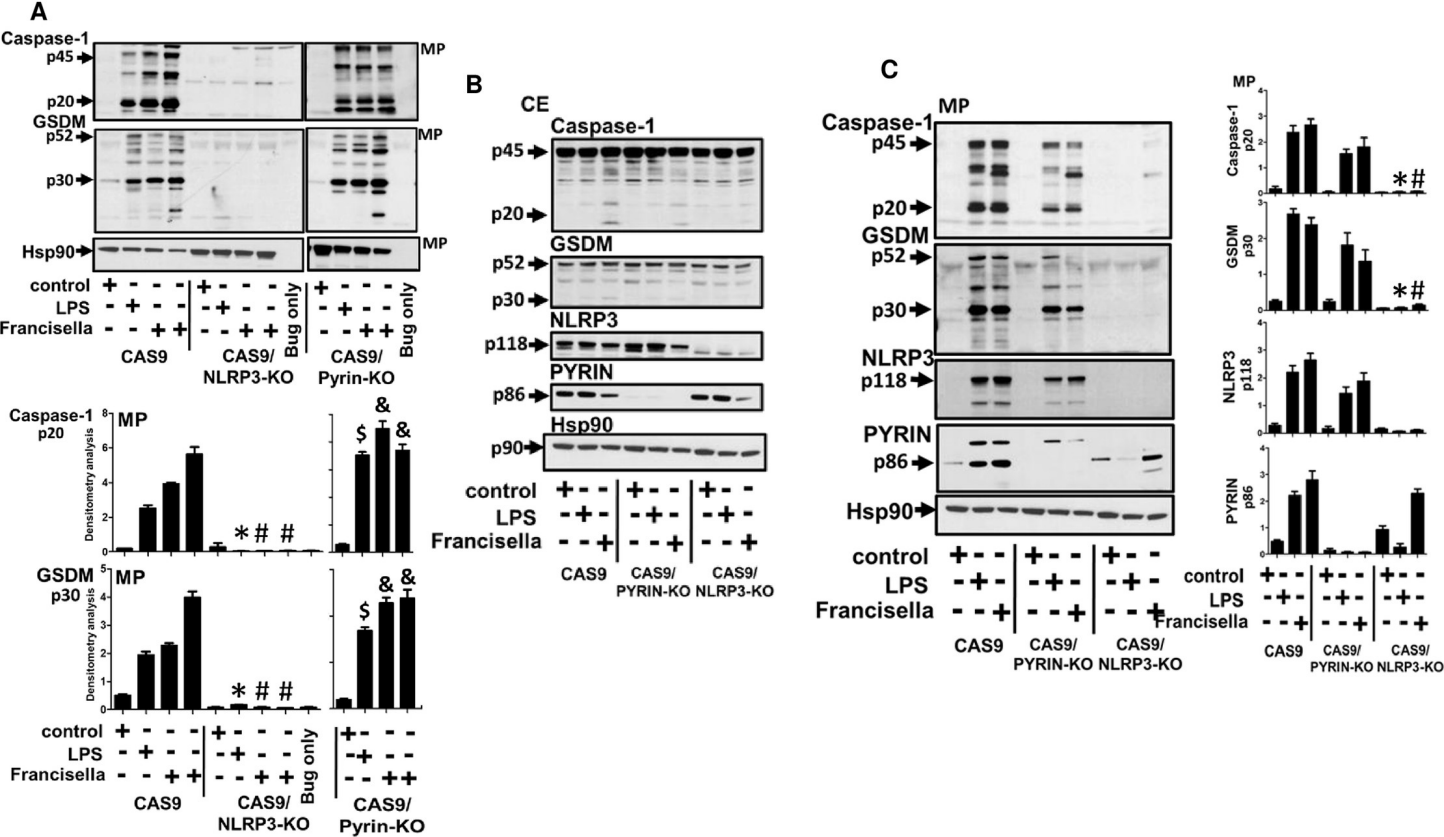
Microparticle caspase-1 and GSDM-D release by *Francisella* regulated by NLRP3 and not Pyrin. A) Microparticles (MP) fractions were isolated from CAS9, CAS9/NLRP3-KO and CAS9/PYRIN-KO cells stimulated with LPS (1μg/ml) for 2h or left untreated, *Francisella* (25MOI) for 2h, 4h or left untreated. Microparticulate p20 caspase-1 and p30 GSDM-D release was abrogated in NLRP3 KO by both LPS and *Francisella*, but not in PYRIN-KO when compared to CAS9 cells. * ^#^ CAS9 vs CAS9/NLRP3 KO; ^$ &^ CAS9/NLRP3 KO vs CAS9/PYRIN KO. B) Cell lysates were analyzed and confirmed for NLRP3 and Pyrin, as well as effects on pro-caspase-1 and pro-GSDM-D. C) Cells were treated with LPS or *Francisella* overnight (17h) and analyzed for the presence of active p20 caspase-1 and cleaved GSDM-D. Overnight experiments were performed to allow internalization of *Francisella* and thereby induce downstream signaling mediated by internalized *Francisella*. * ^#^ CAS9/PYRIN KO vs CAS9/NLRP3 KO. Immunoblot data is representative of n = 4 experiments.

In order to study the signaling cascade regulating inflammasome activation and thereby release of microparticulate active caspase-1 by *Francisella*, we examined the possible roles of NLRP3 vs pyrin as key sensors and signaling regulators of the intracellular pathogen, *Francisella*. CAS9, CAS9/NLRP3-KO, CAS9/PYRIN-KO cells were first stimulated with LPS (1μg/ml) for 4h or left untreated and *Francisella* (25MOI) for 2 and 4h and analyzed for the release of microparticulate active p20 caspase-1 and cleaved GSDM-D. As observed in Fig 1A, microparticulate active p20 caspase-1 and p30 GSDM-D release was completely abrogated in NLRP3 KO by both LPS and *Francisella* (p<0.05), but not in PYRIN-KO when compared to CAS9 cells. This effect was not due to endogenous protein amounts in different cell types, as equal amounts of endogenous pro-caspase-1(p45) and pro-GSDM-D (p52) was detected in the cell lysates (Fig 1B). The abrogation of inflammasome activation by *Francisella* was specific to NLRP3, as knocking down NLRP3 did not impact cytosolic levels of Pyrin (Fig 1B). To determine whether this differential response by *Francisella* was due to external TLR sensing of bacterial LPS during pathogen stimulation via TLR2/TLR4, we stimulated the cells overnight with LPS and *Francisella*. This longer incubation allowed *Francisella* uptake and phagosome escape and hence initiating intracellular inflammasome sensing via its known sensor, pyrin. As seen in Fig 1C, NLRP3-/- cells again completely abrogated release of microparticulate active caspase-1 and cleaved GSDM-D release upon both LPS and *Francisella* stimulation. Interestingly, microparticulate release of active caspase-1 and GSDM-D was not significantly affected in Pyrin -/- cells by *Francisella* or LPS after 17h stimulation when compared to CAS9/NLRP3-/- cells (Fig 1C), similar to our previous short term observation at 2 and 4h stimulations in (Fig 1A). The absence of caspase-1 activity and cleaved GSDM-D observed in NLRP3-/- MPs were not due to MP concentrations (as all lanes were protein and microparticle number normalized, S1A Fig) or due to absence of endogenous p45 caspase-1 or p52 GSDM-D in the cell lysates (Fig 1B). No difference in *Francisella* internalization was also observed between WT, Pyrin -/- or NLRP3 -/- cells (S1B Fig). Although MP encapsulated active caspase-1 and GSDM-D was observed in Pyrin -/- cells when stimulated with LPS and *Francisella*, the amount of microparticulate active caspase-1 and GSDM-D detected was different between early and late stimulation for both LPS and *Francisella*. Compared to CAS9, short time stimulation of 2 and 4h of Pyrin -/- cells showed no difference in microparticulate active caspase-1 and GSDM-D levels. After 17h stimulation the active caspase-1 and GSDM-D were still detected in the released MPs from stimulated Pyrin -/- cells, but the levels were lower than stimulated CAS9. We believe that this was probably due to early enhanced inflammasome activation and active caspase-1 and active GSDM-D release in the absence of Pyrin, leading to increased cell death during longer hours of stimulation. Based on this observation, we hypothesized that Pyrin may be acting as an inhibitor of *Francisella* mediated inflammasome activation and active caspase-1/GSDM-D release, and the absence of pyrin resulted in early elevated levels of microparticulate inflammasome protein release.

### Pyrin overexpression negatively regulated microparticulate active caspase-1 and GSDM-D release by LPS and *Francisella*

**Fig 2:**
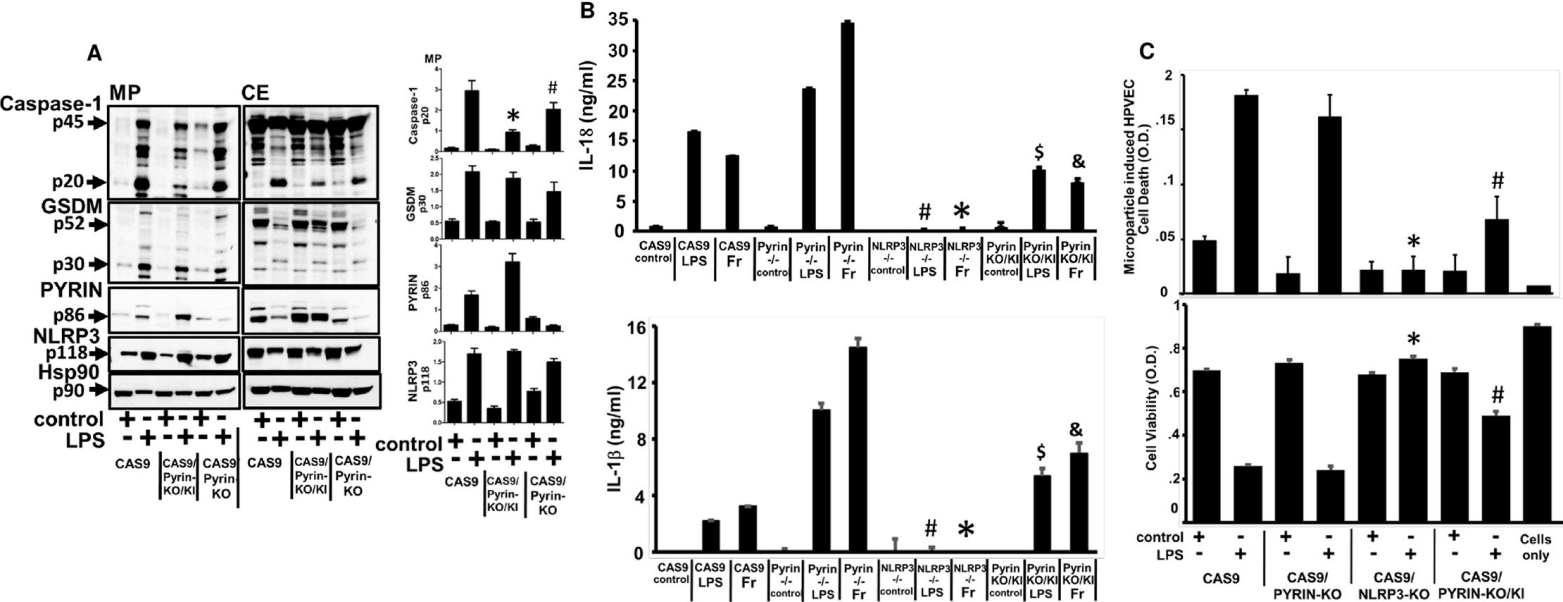
*Francisella* mediated inflammasome responses and cell injury is enhanced in absence of pyrin and reduced upon overexpression of Pyrin in PYRIN-KO/KI cells. A) CAS9, CAS9/PYRIN-KO and CAS9/PYRIN-KO/KI cells were stimulated with LPS (1μg/ml) for 2h or left untreated. Microparticulate p20 caspase-1 and p30 GSDM-D levels were analyzed. p20 active caspase-1 levels in MP- *CAS9 vs CAS9/PYRIN-KO/KI; # CAS9/PYRIN-KO/KI vs CAS9/PYRIN KO. B) CAS9, CAS9/NLRP3-KO, CAS9/PYRIN-KO and CAS9/PYRIN-KO/KI cells were stimulated with LPS (1μg/ml) or *Francisella* for 2h for IL-18; and 17h for IL-1β. Released cytokines were measured in the supernatants from each condition respectively. * ^#^ CAS9 vs CAS9/NLRP3 KO; ^$ &^ CAS9/PYRIN-KO/KI vs CAS9/PYRIN KO C) MPs from CAS9, CAS9/PYRIN-KO, CAS9/NLRP3-KO and CAS9/PYRIN-KO/KI stimulated with LPS (1μg/ml) for 2h or left untreated were subjected to human pulmonary microvascular endothelial cells (HPVEC); co-cultured overnight and analyzed for endothelial cell death by Trypan Blue (cell death assay) and MTS (cell viability assay). Data is representative of n = 4 experiments. * CAS9 vs CAS9/NLRP3 KO; ^#^ CAS9/PYRIN-KO/KI vs CAS9/PYRIN KO.

CAS9 Pyrin KO and Pyrin KO/KI models were compared for the effects on inflammasome activation and active caspase-1/GSDM-D release in microparticles. As seen in Fig 2A, overexpression of Pyrin in CAS9/PYRIN-KO cells (CAS9/PYRIN-KO/KI) significantly inhibited the release of p20 active caspase-1 and active GSDM-D in MPs, as compared to CAS 9 and CAS9/PYRIN-KO cells (p<0.05). Absence of Pyrin in CAS9/Pyrin KO cells, resulted in increased levels of both active caspase-1 and active GSDM-D released in microparticles (p<0.05).This inhibition correlated with higher expression of Pyrin in CAS9/PYRIN-KO/KI cells compared to CAS9 due to overexpression of Pyrin (Fig 2B, right panel); suggesting that Pyrin may act as an inhibitor for microparticulate inflammasome release. Similar effects were also observed in regards to active GSDM-D release.

We measured cytokine release as a measure of caspase-1 activation to confirm our immunoblot observations. We used 2h LPS and *Francisella* stimulation for IL-18; and overnight stimulation for IL-1β release from CAS9, CASP9/Pyrin KO, CAS9/Pyrin KO/KI cells. As seen in Fig 2B, release of both IL-18 and mature IL-1β was significantly abrogated by both LPS and *Francisella* by the Pyrin KO/KI cells (p<0.05) compared to CAS9//Pyrin KO cells. In contrast, as expected from our previous immunoblot observations of presence of active caspase-1, levels of IL-18 and mature IL-1β release from Pyrin KO cells were enhanced compared to CAS9 and CAS9/Pyrin KO/KI cells by both LPS and *Francisella* stimulation (Fig 2B). This cytokine release correlated with levels of active caspase-1 and active p30 GSDM-D levels detected in MPs (Fig 2A).

Our previous studies have demonstrated that upon inflammasome activation monocyte-derived microparticles induce cell death of lymphocytes and lung endothelial cells [9–12]. Hence, MPs from LPS stimulated CAS9, NLRP3-KO, PYRIN-KO and PYRIN-KO/KI were then co-cultured with human pulmonary endothelial cells (HPVEC) overnight and assessed for their ability to induce endothelial cell death. MPs from CAS9 wild type and Pyrin KO cells induced significant cell death, whereas MPs generated from NLRP3 KO cells were unable to induce cell death of HPVEC (p<0.05) (Fig 2C). Interestingly, MPs from overexpressed Pyrin KO/KI cells induced significantly less cell death compared to CAS9 and Pyrin KO cells (p<0.05) (Fig 2C), which correlated with the observed levels of microparticulate active caspase-1 and GSDM-D.

**Fig 3:**
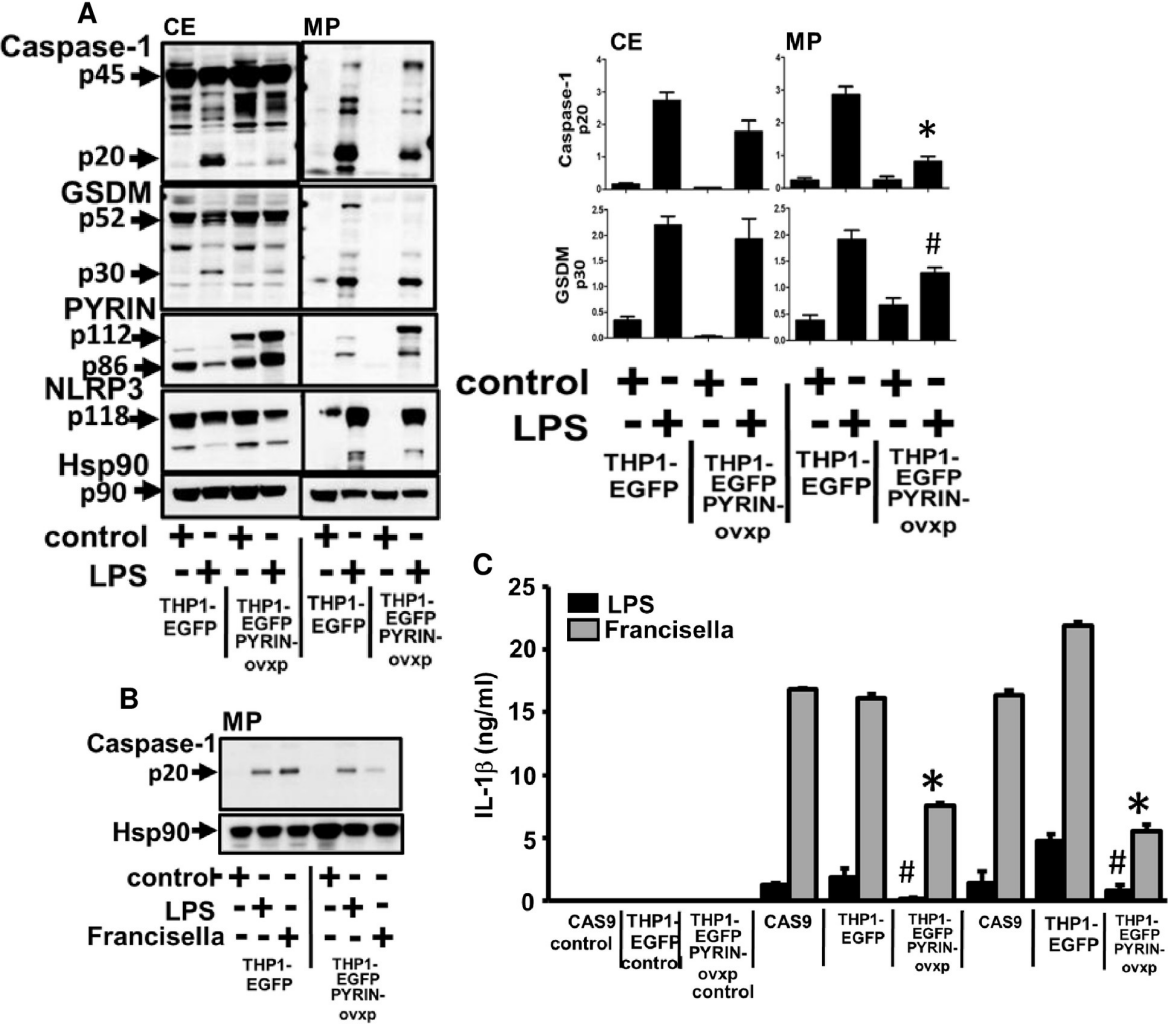
Microparticulate active caspase-1 and GSDM-D release by LPS and *Francisella* is negatively regulated by Pyrin overexpression. A) Cell lysates (CE) and microparticles (MP) fractions were isolated from THP-1 EGFP, THP1-EGFP overexpressed Pyrin cells stimulated with LPS (1μg/ml) for 2h or left untreated and analyzed for the presence of active p20 caspase-1 and p30 GSDM-D. Pyrin-EGFP overexpression was confirmed at 112kD which was not detected in the vector control cells. Endogenous pyrin at 86kD was detected in both cell types. Overexpressing Pyrin significantly inhibited caspase-1 activation (p20) and GSDM-D cleavage (p30) in microparticles. Densitometry scans of active caspase-1 levels from n = 4 experiments. * p20 active caspase-1 levels in MP THP1-EGFP vs THP1-EGFP Pyrin. # p30 active GSDM-D levels in MP THP1-EGFP vs THP1-EGFP Pyrin. B) Similar experiments were performed also with overnight (17h) stimulation with LPS and *Francisella*. THP-1 EGFP, THP1-EGFP overexpressed Pyrin cells were stimulated with LPS (1μg/ml), *Francisella* (25MOI) for 17h or left untreated and analyzed for the presence of active p20 caspase-1 and p30 GSDM-D. C) CAS9, THP-1 EGFP, THP1-EGFP overexpressed Pyrin were stimulated with LPS (1μg/ml) or *Francisella* for 4h, overnight (17h) or left alone. IL-1β was measured from supernatants. Release of IL-1β was significantly diminished with overexpression of Pyrin. Data is representative of n = 4 experiments.THP1-EGFP vs THP1-EGFP Pyrin * IL-1β levels at 4 and 17h upon *Francisella* stimulation; ^#^ IL-1β by LPS stimulation for 4 and 17h.

To further confirm the anti-inflammatory role of Pyrin in microparticulate inflammasome activation and release, we used THP1 cells stably transfected with EGFP or pyrin/EGFP overexpressing Pyrin and stimulated them with LPS for 2h before analyzing active caspase-1 and GSDM-D release. Overexpression of Pyrin significantly reduced caspase-1 activation both in lysates and in microparticles (p<0.05) (Fig 3A). Overnight stimulation with *Francisella* also showed significant inhibition of release of microparticulate active caspase-1(Fig 3B) (p<0.05). When measured for released IL-1β, as expected from our active caspase-1 levels, IL-1β release was significantly abrogated by both LPS and *Francisella* stimulation upon overexpression of Pyrin (THP-1 EGFP Pyrin overexpressed) as compared to vector control (THP-1 EGFP) (p<0.05) (Fig 3C); further supporting that overexpression of Pyrin is acting as an inhibitor for caspase-1 activation and IL-1β release.

## Discussion

Microparticle related inflammasome release represents a novel form of cell-to-cell communication in response to pathogen challenge. Despite remarkable advances in NLRP3 inflammasome responses highlighting the many facets of innate immunity, our understanding of the complexity and critical importance of innate responses in the context of intracellular pathogens upon internalization and microparticle release is rudimentary. Intracellular pathogen, *Francisella* is described upon internalization and escape from phagosome, to be detected by different PRRs, including Pyrin and is thought to trigger inflammasome activation [18–20, 27]. In the current work, we have evaluated the relative role of NLRP3 and pyrin in the microparticle release responses by *Francisella*. Using both exogenous LPS and the intracellular pathogen *Francisella*, we determined that both LPS and *Francisella* mediated microparticle (MP) release and inflammasome responses is regulated by NLRP3 but not pyrin.

Using stable NLRP3 knockout cells, we provide clear evidence that this inflammasome response and release of microparticulate active p20 caspase-1 and p30 GSDM-D is regulated by NLRP3 both for external stimuli LPS, as well as for intracellular pathogen, *Francisella* (Fig 1). This effect on MP release correlated with the subsequent release of caspase-1 dependent IL-1β (17kDa) and IL-18 (18kDa) (Fig 2B). However, we were particularly interested in the pyrin vs. NLRP3 effect on microparticulate release of active caspase-1 or cleaved GSDM-D by either LPS or *Francisella*. Although pyrin has previously been shown to enhance *Francisella* induced cytokine release from primary human monocytes [19, 28], it is also recognized that human cells (THP-1) can recognize extracellular *Francisella* via NLRP3 [18, 28]. Our data clearly confirms NLRP3 as the sensor for intracellular *Francisella* and that absence of pyrin had no effect on sensing and uptake of pathogen and augmented the inflammasome activation by *Francisella* (Figs 1 and 2). MPs from Pyrin-/- cells stimulated with LPS and *Francisella* detected increased active p20 caspase-1 and p30 GSDMD; suggesting that sensing of both LPS and intracellular pathogen, *Francisella* and subsequent release of microparticulate active caspase-1 and GSDM-D is independent of Pyrin (Fig 2).

To further analyze the role of Pyrin in *Francisella* mediated response, we performed similar experiments comparing Pyrin KO and Pyrin KO/KI cells. CAS9/Pyrin KO cells contained increased levels of active caspase-1 and GSDM-D in MPs as compared to LPS MPs from CAS9 cells, which is significantly inhibited upon overexpressing Pyrin in CAS9/ Pyrin KO/KI (Fig 2). This observation correlated with IL-1β levels from supernatant from these cells. We further used stable EGFP overexpression approach to confirm our observation from CAS9 cells. As seen in Fig 3, overexpression of EGFP-Pyrin significantly inhibited both LPS and *Francisella* mediated active p20 caspase-1 and p30 GSDM-D release, which correlated with IL-1β levels in supernatants.

It has been well documented that inappropriate inflammasome activation is involved in many inflammatory diseases [8, 29–33] leading to cell death. Prior work from others as well as our own laboratory had established the novel role of exogenous microparticulate active caspase-1 and cleaved GSDM-D in microparticle-induced apoptosis upon inflammasome activation [9, 10, 11, 34–42]. We and others have recently linked the activation of caspase-1 and GSDM-D protein with vascular cell injury, the central component of acute lung injury (ALI/ARDS) [11, 42]. Intracellular pathogen, *Francisella* is also known to possibly trigger pyroptosome mediated cell death after recognition via pyrin [19, 27]. When comparing the effect of MPs generated in the presence or absence of NLRP3 or pyrin for their ability to induce apoptosis, endothelial cell (HPVEC) apoptosis was only diminished in the NLRP3-/- MPs but not by LPS MPs from CAS9 or Pyrin-/- cells. Cell death induction was significantly lower in LPS MPS from Pyrin KO/KI cells when compared to MPs from CAS9 or Pyrin-/- cells (Fig 2C), which correlated with the amounts of active caspase-1 and cleaved GSDM-D in microparticles.

Our *in vitro* model of stimulation via LPS and *Francisella* was developed to compare the role of NLRP3 and pyrin in regulating inflammasome responses by intracellular pathogen upon internalization and escape from phagosomes. Our results demonstrate that the ability of monocytic THP1 and CAS9, to detect LPS and the intracellular pathogen, *Francisella* is regulated by NLRP3, leading to caspase-1 mediated GSDM-D cleavage and its release in microparticles. We also demonstrate that this regulation via NLRP3 is independent of Pyrin, which has been previously described to be essential for intracellular *Francisella* mediated inflammasome responses [15, 19, 27)]. Our findings are in line with Lagrange *et al*. [18] who also did not observe an impact of MEFV (Pyrin) knockdown on *Francisella* mediated hMDM response, but a major impact of NLRP3 knockdown in promoting downstream IL-1β release. Furthermore, although there are some reports of pyrin deletion which lead to reduced proinflammatory cytokines like IL-1β after *Francisella* stimulation [19], our findings suggest that Pyrin acts as an inhibitor of microparticulate caspase-1 and GSDM-D activation and release by LPS and *Francisella* stimulation. These data suggests an anti-inflammatory role of Pyrin, as described by Chae *et*.*al* and Papin *et*. *al* [22,23] in our model of microparticle release mediated cell injury.

Much remains to be understood about the upstream signaling regulating sensing, activation and MP release and the possible dual role of Pyrin in inflammasome regulation. It will be important to determine the factors regulating sensing by NLRP3 vs Pyrin for LPS or *Francisella*. What happens to NLRP3 upon stimulation? Is this pathway central to other pathogens as well? Is NLRP3 playing an important role in membrane reconstitution and formation of microparticles directly? What regulates the dual role of Pyrin as both pro-inflammatory as well as anti-inflammatory during host pathogen interaction? It is clear that our current novel findings support the need for further studies in understanding the role of NLRP3 in regulating microparticle mediated cell injury and outcomes in inflammatory disease models. Similarly, current studies beyond the scope of this manuscript are also directed towards the signaling cascade regulating the dual role of Pyrin and regulation of inflammasome activation. In summary, the present work indicates that NLRP3 regulates LPS and *Francisella* induced microparticulate caspase-1 activation and GSDM-D mediated pulmonary vascular endothelial cell injury independent of Pyrin.

## Supporting information

S1 Fig A) MPs isolated from CAS9, CAS9/PYRIN KO and CAS9/NLRP3 KO cells treated with LPS (LPS MP) were subjected to quantification analysis for normalization purposes throughtout the experimental procedueres. Total proteins were measured from the MPs. MPs were then also subjected to quantification using the NanoSight technology following the company manual. The Malvern NanoSight range of instruments utilizes Nanoparticle Tracking Analysis (NTA) to characterize nanoparticles from 10nm-2000nm in solution. Based on analysis, LPS MPs from CAS9, CAS9/PYRIN KO and CAS9/NLRP3 KO cells, when normalized using protein quantification showed similar number of particles. Based on this observation, all MPs were analyzed based on protein normalization for experimental purposes of this manuscript. B) *Francisella* uptake was compared between CAS9, CAS9/PYRIN KO and CAS9/NLRP3 KO cells. Briefly, cells were stimulated with GFP tagged *Francisella* at 25 MOI for 17h or GFP vector. Cells were then spun and washed three times with PBS to remove any excess extracellular *Francisella*. Cell lysates were then analyzed for bacterial uptake using GFP antibody. Similar amount of GFP uptake was observed between CAS9, CAS9/PYRIN KO and CAS9/NLRP3 KO cells after 17h.
(TIF)
