# Supplementary figures and images for: Francisella induced microparticulate caspase-1/gasdermin-D activation is regulated by NLRP3 independent of Pyrin
Source: PLoS One. 2018 Dec 31;13(12):e0209931. doi: 10.1371/journal.pone.0209931 (PMC6312237; doi:10.1371/journal.pone.0209931)

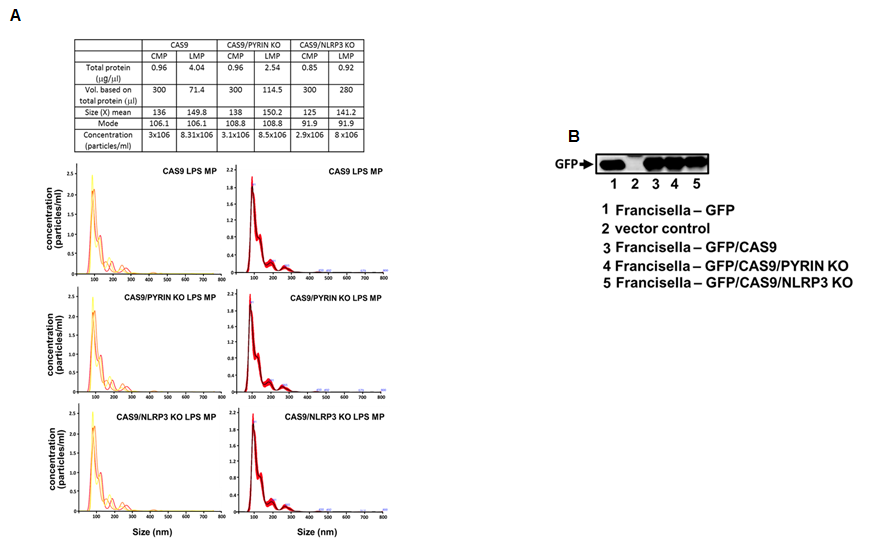

Supplement: S1 Fig — A) MPs isolated from CAS9, CAS9/PYRIN KO and CAS9/NLRP3 KO cells treated with LPS (LPS MP) were subjected to quantification analysis for normalization purposes throughtout the experimental procedueres. Total proteins were measured from the MPs. MPs were then also subjected to quantification using the NanoSight technology following the company manual. The Malvern NanoSight range of instruments utilizes Nanoparticle Tracking Analysis (NTA) to characterize nanoparticles from 10nm-2000nm in solution. Based on analysis, LPS MPs from CAS9, CAS9/PYRIN KO and CAS9/NLRP3 KO cells, when normalized using protein quantification showed similar number of particles. Based on this observation, all MPs were analyzed based on protein normalization for experimental purposes of this manuscript. B) Francisella uptake was compared between CAS9, CAS9/PYRIN KO and CAS9/NLRP3 KO cells. Briefly, cells were stimulated with GFP tagged Francisella at 25 MOI for 17h or GFP vector. Cells were then spun and washed three times with PBS to remove any excess extracellular Francisella. Cell lysates were then analyzed for bacterial uptake using GFP antibody. Similar amount of GFP uptake was observed between CAS9, CAS9/PYRIN KO and CAS9/NLRP3 KO cells after 17h. (TIF) [file pone.0209931.s001.tif]
